# East-West gradient in cardio-vascular mortality in Austria: how much can we explain by following the pattern of risk factors?

**DOI:** 10.1186/1476-072X-10-59

**Published:** 2011-11-14

**Authors:** Katharina V Stein, Anita Rieder, Thomas E Dorner

**Affiliations:** 1 Institute of Social Medicine, Centre for Public Health, Medical University of Vienna, Vienna, Austria

## Abstract

**Background:**

Various studies show major regional differences in the prevalence of cardio-vascular disease morbidity and mortality, both in Europe and within European countries. In Austria, these differences are documented by an East-West gradient with declining morbidity and mortality rates when moving from the East to the West of the country. It was the aim of this study to analyse if, and to what extent, socio-demographic and socio-economic determinants, social resources and health behaviour can contribute to the clarification of this East-West gradient by conducting secondary analyses of an existing Austrian health dataset.

**Results:**

The data were analysed using bivariate analyses, as well as univariate and multivariate logistic regression models. These analyses revealed significant East-West gradients for various risk factors, as well as socio-demographic and socio-economic health determinants. There was a gradual decrease of hypertension, diabetes mellitus, obesity, and psycho-social discomfort in both sexes, with the highest prevalences in those Austrian regions with the highest cardio-vascular mortality and a stepwise decrease to the regions with the lowest cardio-vascular mortality. Controlling for educational level significantly raised the odds for diabetes, hypertension and obesity. In the results of the multivariate analyses, factors that significantly and independently predicted diabetes mellitus were geographic location, psycho-social discomfort, lack of physical exercise, and age in both sexes. For women these factors additionally included a low educational level, lack of social support, and being born abroad.

**Conclusions:**

Our study shows a clear gradual decline of cardio-vascular mortality and some of its risk factors from East to West in Austria. Concerning these risk factors, the geographic region and psycho-social discomfort showed the greatest association with diabetes mellitus, hypertension, and obesity. Hence, they contribute to the explanation of the variance in spatial cardio-vascular disease mortality. Yet, a large proportion of this variance remains unexplained. It would be of great importance to public health and preventive measures to take a closer look at spatial differences in cardio-vascular disease morbidity and mortality to better tailor programmes to the regional environments and settings. Our results also call for a greater importance of preventative measures for psycho-social discomfort and increase of social support.

## Introduction

In Europe, major differences in geographic regions regarding mortality from cardio-vascular diseases (CVD) have been observed with a high cardio-vascular mortality in the eastern and north-eastern European countries and a lower CVD mortality in western and south-western European countries [1-3]. An East-West gradient in cardio-vascular mortality has also been reported for countries within Europe like Germany [4], and France [5]. Austria, a small country in the centre of Europe, has a length of 580 km from the most eastern to the most western point. Despite the relative small size, remarkable differences in cardio-vascular epidemiology have been reported in previous investigations [6].

One way of explaining the significant variance in CVD prevalence and mortality is by taking a closer look at the risk factors. A large proportion of these risk factors are modifiable [7], hence the variance in CVD morbidity and mortality may be explained by analysing the distribution of these risk factors. In fact, as causes for the regional differences in CVD mortality in Europe, differences in coronary risk factors like smoking, hypertension, dyslipidemia, diabetes mellitus, overweight as well as socio-economic factors, lifestyle variables, medical care, genetic factors, and environmental conditions have been identified [3,8]. For Austria, a similar east-west gradient has been reported for CVD mortality as for cardio-vascular risk factors like diabetes mellitus, obesity, or lack of physical exercise [9-12].

It was the aim of this study to analyse if, and to what extent socio-demographic and socio-economic determinants, social resources and health behaviour can contribute to the clarification of an east-west gradient in CVD mortality in Austria by conducting secondary analyses of an existing health dataset.

### A theoretical model on health determinants for CVD

**Figure 1:**
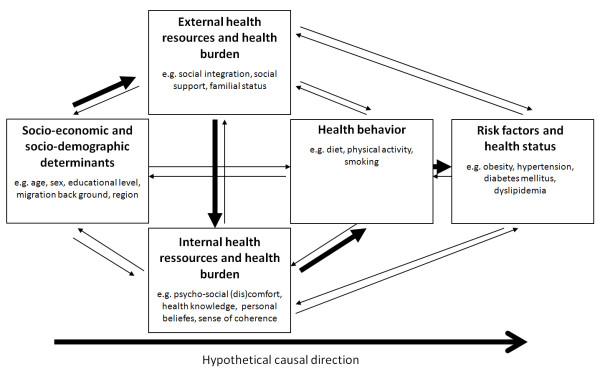
**Main theory for the development of cardiovascular diseases based on health determinants, modified from: **[14,15].

Health in general and cardio-vascular health in particular is determined by multiple factors, most of which are grounded in the living conditions and environment of an individual. Health is greatly influenced by demands and resources of the external and internal environments an individual is exposed to [13,14]. Figure 1 illustrates the complex interrelationships between socio-economic and socio-demographic determinants on the one side (e.g. age, sex, or geographical origin), with health behaviour and, ultimately, individual risk factors and health status on the other side. All of these domains are further interlinked with external (e.g. social integration or emotional support) and internal health resources (e.g. health locus of control or sense of coherence) [14,15]. For CVD, not only the classical risk factors hypertension, dyslipidemia, diabetes mellitus, obesity, and smoking [16,17], or lifestyle associated risk factors like a calorie-rich diet with low intake of fruits and vegetables, and lack of exercise [7] have been identified as independent risk factors. Various authors have also shown, that psycho-social factors like depression and anxiety, chronic life stress, or lack of social support [18,9] and socio-economic or socio-demographic factors like low education and income, or the geographic origin and current location [19,20] are relevant health determinants and independent risk factors for CVD. All of these vary in the different regions of Europe but also within one country like Austria, and so, they may play a role in the explanation of the variance in CVD mortality across and within countries.

## Methods

Mortality data for men and women in age groups of ten year intervals in each of the nine Austrian NUTS-2 regions as well as the number of inhabitants with that age in each region for the years 2003 to 2009 were obtained by the respective official year books from Statistics Austria, the national statistics agency. Regarding disease-specific mortality, numbers of death due to CVD (ICD-10 codes I00-I99) were obtained. With this crude dataset cardiovascular mortality rates were computed per 100,000 inhabitants for all Austrian regions. The data were age-adjusted according to the Austrian population for 2009. For the seven analysed years, furthermore the mean was computed. Based on these sex-specific mean mortality data, Austria was divided into four regions (region 1 - region 4) with decreasing mortality rates from region 1 to region 4. Regions 1 and 2 had higher mortality rates than the Austrian mean and regions 3 and 4 lower mortality rates. The data were divided into four regions to be able to show the stepwise regression of mortality rates. This would not have been achieved with choosing regions. On the other side, more regions would not have allowed for a sufficient number of respondents in each region.

### Database

The database used for the analysis of cardio-vascular risk factors was the Austrian Health Interview Survey (AT-HIS) 2006-07 [21]. The survey had been commissioned by the Austrian Federal Ministry of Health, Family and Youth and was carried out by Statistics Austria. The sample was stratified by geographic region, with equal numbers of subjects being included in each region. The subjects were interviewed between March 2006 and March 2007 by a total of 137 specially trained interviewers. The gross sample size was 25,130 people older than 15 years. The response rate after excluding the neutral deficiency was 63.1%. The interviews were conducted face-to-face using CAPI (computer assisted personal interviewing). The questionnaire included 450 questions regarding diseases and complaints, subjective health status, health behaviour, quality of life, and health care utilisation, as well as socio-demographic and socio-economic variables. It was designed based on the European Core Health Interview Survey (EC-HIS) [22] and was adapted to Austrian requirements by an expert panel. In order to account for the stratification of the sample, the data were weighted by geographic region, age, and sex.

### Overview of questions and parameters utilised from AT-HIS

Regarding the diagnoses diabetes mellitus and hypertension the subjects were asked if they ever had been diagnosed with diabetes mellitus or high blood pressure, respectively.

Obesity was defined as having a body mass index of 30 kg/m^2 ^or higher, which was computed using self-reported data of body weight and body height.

Smoking was given when subjects answered "yes, daily" to the question "Do you smoke currently?".

Regarding nutrition, the subjects were asked "How would you describe your eating habits?". The possible answers were "mixed diet with many fruits and vegetables", "mixed diet low in meat", "mixed diet rich in meat", "vegetarian with dairy products and/or eggs", "vegetarian, with fish and/or dairy products and/or eggs", and "vegetarian and no animal products". The variable was dichotomized with a mixed diet rich in meat vs. all other possible diets.

Physical activity was assessed using the question "Do you sweat at least once a week during your leisure time due to physical activity? E.g. by running, cycling, aerobics, etc.?" and the question "On how many days per week on average?". A lack of physical exercise was defined if a person indicated to sweat less than four times a week due to exercise.

Psycho-social discomfort was described with the questions "How often during the last four weeks did you feel ..." "nervous", "abject", "sad", "exhausted" and "tired". Possible answers were "always", "mostly", "quite often", "sometimes", and "never". If a subject indicated that at least one of the dimensions of discomfort had occurred "always" or "mostly" they were classified as suffering from psycho-social discomfort. This indicator, which was represented with those five items, showed a reliability with Cronbach's alpha of 0.807.

Regarding social support subjects were asked "How satisfied are you with the support you get from your friends?". Possible answers were "very satisfied", "satisfied", "neither satisfied nor dissatisfied", "dissatisfied", and "very dissatisfied". If a person indicated "very dissatisfied" or "dissatisfied" he or she was classified to be lacking social support.

Low education was defined as any education without "Matura", the Austrian high school graduation exam, which is usually taken after a total of 12 or 13 years of primary and secondary education at ages 18 or 19 and which is the prerequisite for continuing to tertiary education.

Migration status was mirrored in the question "In which country were you born?". Possible answers were "Austria" and "in any other country".

Marital status was represented with the question "What is your current marital status?". Possible answers were "unmarried", "married and living with the partner", "married and living separate", "widowed", and "divorced". The variable was dichotomized as "Married and living with a partner" vs. "Not married and not living with a partner", which includes all other possibilities.

Age was assessed in groups of 5 year intervals.

### Statistical analysis

For statistical analysis SPSS Statistics 17.0 was used. Bivariate analyses were undertaken by means of cross-tabs, and group differences were assessed with the Pearson's Chi^2^-test and the Linear-by-Linear Association. Univariate logistic regression models were computed with diabetes mellitus, hypertension, and obesity as the dependent variables and the 4 Austrian regions with different cardio-vascular mortality as the categorical independent variable. In this analysis, the Austrian region with the lowest cardio-vascular mortality was defined as the reference group. In the next step, we calculated logistic regression models for diabetes mellitus, hypertension, and obesity as dependent variables, and the 4 Austrian regions as independent variable and added each of the following control variables separately to the model: age, smoking status, diet rich in meat, lack of physical exercise, psycho-social discomfort, lack of social support, low educational level, being born abroad, and not being married and not living with a partner. In these models, the change of odds ratios for the dependent variables by regions could be observed through controlling for each of the variables. Furthermore, a multivariate logistic regression analysis was performed, and again diabetes mellitus and hypertension were defined as dependent variables. All independent variables (regions with different cardio-vascular mortality, age, obesity vs. non-obesity, smoking vs. not currently smoking, diet rich in meat vs. any other kind of diet, lack of physical exercise vs. exercising at least four times a week, psycho-social discomfort vs. no psycho-social discomfort, lack of social support vs. good social support, low education vs. education at least up to the "Matura", being born abroad vs. being born in Austria, and being married and living with partner vs. any other kind of marital status) were included in the model simultaneously. All independent variables except age were classified as categorical variables; age was classified as continuous variable with a 5 years interval. The results of all logistic regression models are represented as odds ratios with 95% confidence intervals. Nagelkerkes' R^2 ^is presented as a measure of model-fit. All results are stratified by sex.

### Ethical considerations

The secondary analysis for this study was approved by the Ethics Committee of the Medical University Vienna (EC # 770/2011).

## Results

**Figure 2:**
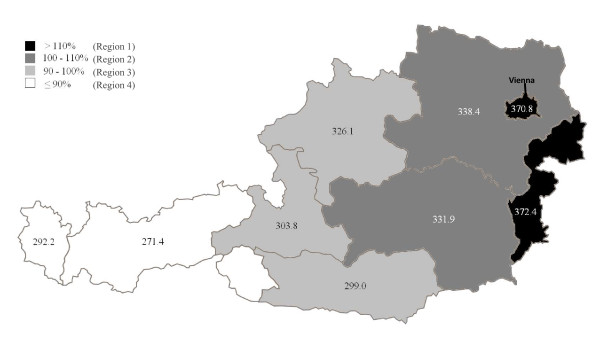
**Mean mortality rates (2003-2009) among male subjects in Austrian provinces, age adjusted according to the male Austrian population 2009**. 100% represents the mean mortality rate in Austria. Dark shadings indicate regions with mortality rates above and light shadings below the Austrian mean. Numbers indicate mean mortality rates/100.000.

**Figure 3:**
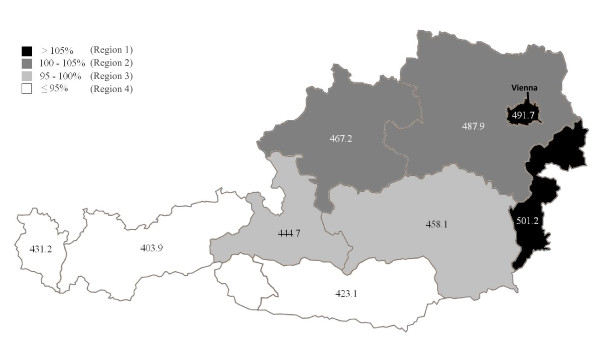
**Mean mortality rates (2003-2009) among female subjects in Austrian provinces, age adjusted according to the female Austrian population 2009**. 100% represents the mean mortality rate in Austria. Dark shadings indicate regions with mortality rates above and light shadings below the Austrian mean. Numbers indicate mean mortality rates/100.000.

Crude mean CVD mortality rates of the years 2003-2009 varied between 405.9/100.00 in the very eastern and 250.6/100.000 in the very western Austrian NUTS-2 regions in men and between 536.5 and 333.1/100.000 in women. Mean age-adjusted mortality rates varied between 372.4 and 271.4/100,000 in men and between 501.2 and 403.9/100.000 in women. The mean age-adjusted cardiovascular mortality rate in Austria was 330.2 (SD = 7.2)/100,000 for men and 464.6 (SD = 20.3)/100,000 for women. CVD mortality rates gradually decreased from the eastern to the western Austrian NUTS-2 regions. Age-adjusted CVD mortality rates for each NUTS-2 region are shown in Figure 2 for men and in Figure 3 for women. When compared with the mean CVD mortality for all of Austria, the NUTS-2 regions can be grouped into four regions with different cardio-vascular mortality rates (regions 1 to 4). For both, men and women the highest CVD mortality rates were found in the very eastern Austrian NUTS-2 region and in the capital of Austria, Vienna. Those were grouped to region 1. Regions 2 to 4 showed a continuous decline in CVD mortality rates from the east to the west in men (Figure 2) and from north-east to south-west in women (Figure 3) compared to this region 1. This classification into four regions was hence used for further analyses of CVD determinants.

### Results by risk factors

As shown in tables 1 and 2, there was a gradual decrease of the proportion of hypertension, diabetes mellitus, obesity, and psycho-social discomfort in both sexes, with the highest prevalences in the Austrian regions with the highest cardio-vascular mortality and a stepwise decrease to the regions with the lowest cardio-vascular mortality. In both sexes, lack of physical exercise, lack of social support, and in women, additionally a diet rich in meat, was also highest in region 1 and decreased stepwise to region 3, but rose again to a higher prevalence in region 4. Regarding educational level, the gradient depicted a counter-directional development, with the highest proportion of men and women with low educational level in the region with the lowest CVD mortality (region 4), increasing stepwise to the region with the highest CVD mortality (region 1). The proportion of male and female smokers was high in all regions. The proportion of men and women, who were born abroad, peaked in the region with the highest CVD mortality, but was lowest in region 2 and increased stepwise to region 4. There were also significant differences in the four Austrian regions regarding the proportion of subjects not being married and not living with a partner. However, within this variable, no clear east-west gradient was observable.

**Table 1:** Prevalence of cardio-vascular risk factors in different Austrian regions (east - west): Results for men in %.

	Region 1*	Region 2*	Region 3*	Region 4*	P**	P***
N	1741	2533	2233	947		

Diabetes mellitus	7.9	5.3	4.1	4.4	< 0.001	< 0.001
Hypertension	20.1	22.0	19.0	16.6	0.002	0.009
Obesity	13.4	12.7	11.2	10.0	0.025	0.003
Smoking	30.8	25.1	25.6	30.8	< 0.001	0.378
Diet rich in meat	37.9	41.6	40.8	40.3	0.096	0.196
Lack of physical exercise	73.9	67.9	64.9	68.0	< 0.001	< 0.001
Psycho-social discomfort	14.2	10.4	8.9	7.8	< 0.001	< 0.001
Lack of social support	4.2	2.3	1.9	3.0	< 0.001	0.004
Low educational level	62.1	75.5	77.8	79.8	< 0.001	< 0.001
Born abroad	30.0	9.9	12.7	14.7	< 0.001	< 0.001
Not being married	45.7	40.7	41.2	42.1	0.007	0.040
Age older than 60 years	22.3	24.1	22.5	21.3	0.283	0.435

*Regions 1-4 are depicted in figure 2 for men

**Table 2:** Prevalence of cardio-vascular risk factors in different Austrian regions (east - west): Results for women in %.

	Region 1*	Region 2*	Region 3*	Region 4*	P**	P***
N	1934	2854	1683	1550		

Diabetes mellitus	7.2	6.7	6.1	5.3	0.111	0.015
Hypertension	25.6	22.5	21.7	19.4	< 0.001	< 0.001
Obesity	14.0	14.6	10.3	10.4	< 0.001	< 0.001
Smoking	19.8	19.7	17.2	20.5	0.073	0.795
Diet rich in meat	16.5	13.7	12.2	14.6	0.002	0.045
Lack of physical exercise	81.6	75.1	72.8	77.9	< 0.001	0.002
Psycho-social discomfort	15.7	11.1	13.0	10.9	< 0.001	0.001
Lack of social support	4.8	2.9	2.3	2.6	< 0.001	< 0.001
Low educational level	65.6	78.5	77.5	79.8	< 0.001	< 0.001
Born abroad	25.9	11.5	11.4	15.0	< 0.001	< 0.001
Not being married	52.6	46.3	48.4	50.9	< 0.001	0.615
Age older than 60 years	29.3	29.4	29.4	27.8	0.681	0.367

*Regions 1-4 are depicted in figure 3 for women;

### Results by unadjusted odds ratios

In tables 3, 4, and 5 the unadjusted odds ratios are shown for suffering from diabetes mellitus, hypertension or obesity in the four different Austrian regions. These underline clear differences in the prevalence of these three conditions. Adjustments for psycho-social discomfort and for lack of physical exercise greatly decrease the chance of being affected by diabetes mellitus, hypertension, and obesity in regions 1 to 3 compared to region 4 (results not shown) while the adjustment for lack of social support did so to a lesser extent. Hence it can be concluded that these factors contribute to the explanation of the geographic variance of the analysed cardio-vascular risk factors.

**Table 3:** Influence of region on the risk of diabetes mellitus; results of logistic regression models, unadjusted and adjusted for a variety of co-factors

	Men			Women		
	**OR**	**95% CI**	**P**	**OR**	**95% CI**	**P**

Univariate logistic regression models						

Region 3*	0.92	0.63-1.34	0.672	1.16	0.86-1.57	0.331
Region 2*	1.21	0.85-1.73	0.282	1.30	0.99-1.69	0.056
Region 1*	1.86	1.31-2.66	0.001	1.39	1.05-1.85	0.021

Multivariate logistic regression models						

Region 3*	0.88	0.59-1.30	0.504	1.13	0.82-1.55	0.450
Region 2*	1.10	0.76-1.60	0.610	1.29	0.97-1.71	0.076
Region 1*	1.84	1.26-2.69	0.002	1.36	1.01-1.84	0.043
Co-Variates						
Age	1.39	1.34-1.44	< 0.001	1.31	1.27-1.35	< 0.001
Smoking (yes)	1.02	0.77-1.36	0.881	1.01	0.74-1.38	0.959
Diet rich in meat (yes)	1.01	0.80-1.28	0.936	0.78	0.57-1.06	0.111
Lack of physical exercise (yes)	1.46	1.10-1.93	0.008	1.33	1.00-1.77	0.047
Psycho-social discomfort (yes)	1.82	1.38-2.40	< 0.001	1.87	1.49-2.35	< 0.001
Lack of social support (yes)	1.58	0.97-2.56	0.067	1.79	1.23-2.58	0.002
Low educational level (yes)	0.97	0.75-1.26	0.837	2.00	1.44-2.78	< 0.001
Born abroad (yes)	0.82	0.64-1.06	0.238	1.42	1.09-1.84	0.009
Not being married (yes)	0.82	0.64-1.06	0.132	1.03	0.85-1.26	0.740
R^2^	0.216			0.204		

*Region 4 is used as a reference region; regions 1-4 are depicted in figures 2 for men and 3 for women

**Table 4:** Influence of region on the risk of hypertension; results of logistic regression models, unadjusted and adjusted for a variety of co-factors

	Men			Women		
	**OR**	**95% CI**	**P**	**OR**	**95% CI**	**P**

Univariate logistic regression models						

Region 3*	1.19	0.97-1.45	0.095	1.15	0.97-1.36	0.109
Region 2*	1.42	1.17-1.73	< 0.001	1.21	1.03-1.41	0.017
Region 1*	1.27	1.03-1.56	0.024	1.43	1.22-1.68	< 0.001

Multivariate logistic regression models						

Region 3*	1.16	0.93-1.44	0.196	1.13	0.93-1.38	0.209
Region 2*	1.36	1.09-1.68	0.005	1.22	1.02-1.45	0.028
Region 1*	1.31	1.04-1.65	0.023	1.58	1.31-1.91	< 0.001
Co-Variates						
Age	1.33	1.30-1.36	< 0.001	1.37	1.35-1.40	< 0.001
Smoking (yes)	0.93	0.80-1.09	0.387	0.95	0.80-1.14	0.953
Diet rich in meat (yes)	1.02	0.90-1.17	0.727	1.36	1.14-1.61	0.001
Lack of physical exercise (yes)	1.05	0.91-1.21	0.523	0.92	0.79-1.07	0.290
Psycho-social discomfort (yes)	1.47	1.22-1.79	< 0.001	1.61	1.36-1.90	< 0.001
Lack of social support (yes)	1.55	1.10-2.19	0.013	0.93	0.68-1.27	0.644
Low educational level (yes)	1.14	0.97-1.33	0.104	2.03	1.70-2.43	< 0.001
Born abroad (yes)	0.80	0.67-0.97	0.021	1.43	1.21-1.70	< 0.001
Not being married (yes)	0.77	0.66-0.89	< 0.001	0.78	0.69-0.89	< 0.001
R^2^	0.247			0.319		

*Region 4 is used as a reference region; regions 1-4 are depicted in figures 2 for men and 3 for women

**Table 5:** Influence of region on the risk of obesity; results of logistic regression models, unadjusted and adjusted for a variety of co-factors

	Men			Women		
	**OR**	**95% CI**	**P**	**OR**	**95% CI**	**P**

Univariate logistic regression models						

Region 3*	1.14	0.89-1.46	0.316	0.99	0.79-1.24	0.919
Region 2*	1.31	1.03-1.67	0.030	1.47	1.21-1.79	< 0.001
Region 1*	1.40	1.09-1.81	0.009	1.41	1.14-1.73	0.001

Multivariate logistic regression models						

Region 3*	1.13	0.88-1.46	0.350	1.00	0.79-1.26	0.991
Region 2*	1.29	1.01-1.65	0.044	1.50	1.23-1.82	< 0.001
Region 1*	1.50	1.15-1.95	0.002	1.42	1.14-1.76	0.001
Co-Variates						
Age	1.10	1.07-1.12	< 0.001	1.11	1.09-1.13	< 0.001
Smoking (yes)	0.84	0.71-1.00	0.053	0.99	0.79-1.26	0.991
Diet rich in meat (yes)	1.61	1.39-1.87	< 0.001	1.90	1.60-2.26	< 0.001
Lack of physical exercise (yes)	1.18	1.00-1.40	0.046	1.10	0.92-1.30	0.301
Psycho-social discomfort (yes)	1.17	0.94-1.46	0.150	1.49	1.24-1.78	< 0.001
Lack of social support (yes)	1.30	0.88-1.91	0.185	0.99	0.70-1.41	0.972
Low educational level (yes)	1.81	1.49-2.19	< 0.001	1.94	1.59-2.37	< 0.001
Born abroad (yes)	1.13	0.93-1.38	0.220	1.36	1.14-1.63	0.001
Not being married (yes)	0.73	0.62-0.86	0.132	0.87	0.76-1.00	0.046
R^2^	0.062			0.080		

*Region 4 is used as a reference region; regions 1-4 are depicted in figures 2 for men and 3 for women

Further analysis brought to light that controlling for educational level significantly raised the odds for diabetes, hypertension and obesity (results not shown), which leads to the conclusion that, if educational levels were equal in all Austrian regions, the east-west gradient in diabetes mellitus, hypertension, and obesity would be even more pronounced.

### Results by disease

In tables 3, 4, and 5, the results of multivariate analyses are presented, in which all independent variables are included in the model simultaneously. Apart from the geographic location (region) other co-factors that significantly and independently predicted diabetes mellitus were psycho-social discomfort, lack of physical exercise, and age in both sexes. For women these factors additionally included a low educational level, lack of social support, and being born abroad.

For hypertension, independent predictors were lack of social support, psycho-social discomfort, and age again for both sexes. These were supplemented by a lack of social support for men, and a low educational level, being born abroad, and a diet rich in meat for women. On the other side, not being married acted as a protective factor for both sexes, and being born abroad further influenced the risk positively for men.

Concerning obesity, a low educational level, a diet rich in meat, and age functioned as independent predictors in both sexes, added by a lack of physical exercise in men, and psycho-social discomfort, and being born abroad in women. Again, not being married was a protective factor in both sexes.

## Discussion

Our results show a remarkable east-west decline in cardiovascular mortality in Austria, with mortality rates being elevated by 62% for men and 61% for women, when comparing the eastern-most with the western-most Austrian NUTS-2 region. A clear gradual decline from east to west is also found for the CVD risk factors diabetes mellitus, hypertension, obesity, a diet rich in meat, lack of physical exercise, psycho-social discomfort, and lack of social support. Thus, these risk factors will obviously contribute to the explanation of the east-west gradient for CVD mortality in Austria. A low educational level showed a west-east gradient (counter-directional) and hence contributes to smoothing the east-west gradient in CVD mortality. The multivariate regression model revealed that adjustment for different co-variates which independently predicted diabetes mellitus, hypertension and obesity, did not influence the odds ratios for these three conditions in the 4 analyzed regions.

### European dimension

This Austrian East-West gradient continues over the national borders. Its Eastern neighbour, Hungary, has one of the highest CVD mortality rates within Europe, and its Western neighbouring country, Switzerland, has one of the lowest [3]. For variance of CVD morbidity and mortality between the European countries, variation in classical risk factors like hypertension, hyperlipidemia, and overweight, risk behaviour like smoking, physical activity, and nutritional habits, as well as variance in socio-economic variables, psycho-social and environmental factors, or medical care, all have been discussed to be responsible [3]. The Seven Countries Study, which was performed in five European countries, the USA, and Japan revealed dietary intake of saturated fat, smoking, serum cholesterol, and hypertension to be major factors for the spacial variability in CVD morbidity and mortality [23,24]. In the European MONICA (Monitoring of Trends and Determinants In Cardio-Vascular Disease) project, a trend over time of declining smoking rates, mean blood pressure, and cholesterol concentration led to a decreased CVD morbidity, despite increase in BMI [25].

### Explaining the spatial differences

Our findings demonstrated that self-reported prevalence of diabetes mellitus, hypertension, and obesity follow the same spatial patterns as CVD mortality. A minor part of this variance can be explained by age, health behaviour, psycho-social and socio-economic factors. As shown by the R^2^, 6 to 32% of the variance in diabetes mellitus, hypertension and obesity could be explained through the regression analyses, when all co-variates were included. Through controlling for the co-variates physical exercise, psycho-social discomfort, and lack of social support the geographical effect of the region on odds for diabetes mellitus, hypertension and obesity decreased. These results support the conclusion that those factors contributed to the explanation of the spatial differences in diabetes mellitus, hypertension and obesity prevalence.

In our multivariate regression model, region was one of the strongest predictors for diabetes mellitus, hypertension, and obesity. In some analyses, geographical location was even a stronger explanatory factor than age, lifestyle factors, psycho-social discomfort, or socio-economic factors. Apart from region, psycho-social discomfort was the strongest predictor for cardiovascular risk factors. Psycho-social discomfort [26,27], lack of social support [26,28] and a low socio-economic status [26,29,30] have all been identified in previous studies to elevate the risk for CVD. Since there is a clear East-West gradient in our study concerning subjects who indicated psycho-social discomfort, and this is one of the strongest predictors of diabetes mellitus and hypertension, it is likely that psycho-social discomfort greatly contributes to the explanation of the regional differences in cardio-vascular mortality.

Regarding lifestyle factors, a lack of physical exercise was a significant predictor for diabetes mellitus in both sexes and for obesity in women. Additionally, a diet rich in meat was significantly associated with obesity in both sexes and with hypertension in women. The geographic East-West gradient in the lack of physical exercise and obesity, demonstrated by our data, is in line with previous Austrian studies [10-12].

Not being married and not living with a partner was a protective factor for hypertension in both sexes and for obesity in women. This finding is in contradiction with results from other studies, where not being married was associated with a greater prevalence of cardio-vascular risk factors or cardio-vascular mortality [31-33].

Migrational background, which showed a great variability in the different Austrian regions, was a significant independent predictor for diabetes mellitus, hypertension, and obesity in women only, and a protective factor for hypertension in men. Migration effects have already been discussed to be an influencing factor on CVD mortality and East-West-differences in Europe [1].

Returning to our theoretical model depicted in Figure 1, our findings support the significant influence of the models' propositioned health determinants, especially the socio-demographic variables region and migration background, the external health resource social support, the internal health resource psycho-social comfort and the health behavioural indicators diet, smoking and physical activity. Our data furthermore illustrate that not every determinant has the same effect on either men and women or on the health outcomes diabetes mellitus, hypertension and obesity. Thus, the merit of the model lies primarily in summarising the complex interactions of the modifiable and given health determinants, while the suggested directionality remains to be proven. Further investigations are also needed to quantify the contributions of the different health determinants to the various health outcomes.

## Conclusions

Based on the model of interrelationships between socio-economic determinants, external and internal health resources, health behaviour and health status (see Figure 1), we examined a number of socio-economic, psycho-social, and behavioural factors and their association with the classical CVD risk factors diabetes mellitus, hypertension, and obesity. Our variables match with the health determinants specified and the indicators outlined in each of the domains. However, we could not include all relevant variables into our regression model and these could only explain some part of the spatial variance in risk factors and hence, in CVD mortality. This constitutes the main limitation of our study, since we still cannot explain the whole magnitude of the East-West gradient.

Hence, additional explanations for the great variance in CVD mortality by region must be found and analyzed. The most apparent difference between the eastern and the western parts of Austria is their different geographic appearance, with the central Alps in the West and the beginning of the Pannonian lowlands in the East. This goes hand in hand with distinct socio-cultural and socio-historic developments. To what extent these elements influence CVD mortality needs further exploration. Resulting regional differences regarding attitudes and values would also require further investigation. For example, it is said that people in Eastern Austria indulge in a diet rich in sugar and fat (which can partly be explained by social-cultural developments), whereas people in Western Austria are much more active indoors and outdoors, and physical exercise in general has a high priority among all age groups. Location itself has previously been discussed to be an independent contributing factor towards health. Theoretical models explaining the significance of geographical location (and climate) in the production and maintenance of health variations regard it to be relevant because it constitutes and contains social relations and physical resources [34,35]. A similar approach is taken by eco-epidemiology, where social environments and communities are analysed and used to explain risk factors for chronic diseases, such as CVD [36]. However, it has been argued that region cannot be regarded independently from all other potential contributing factors and that focusing on location as an independent contributing factor towards health inequalities may lead to an underestimation of the contribution of it to disease risk [35].

From our data, certainly no causal relationship between the analysed classical risk factors diabetes mellitus, hypertension, and obesity, behavioural and social risk factors and CVD mortality can be derived, as proposed by the theoretical model (Figure 1). However, we could demonstrate a significant influence of regional location on some of the socio-economic, external and internal health resources and health behaviours analysed. Nevertheless it goes without mentioning that we assessed CVD mortality and CVD risk factors simultaneously, however it takes years or decades before changes in risk factors will be visible in CVD mortality.

In conclusion, our study shows a clear gradual decline of CVD mortality from Eastern to Western regions in Austria. Such a gradual East-West decline was also found for the CVD risk factors diabetes mellitus, hypertension, obesity, a diet rich in meat, lack of physical exercise, psycho-social discomfort and lack of social support. Apart from the region, psycho-social discomfort showed the greatest association with diabetes mellitus, hypertension, and obesity hence it most likely contributes to spacial CVD mortality variance to a great extent. This result would also call for bigger efforts in preventative measures targeted at coping with psycho-social discomfort and increase of social support when considering cardio-vascular mortality and morbidity.

Although some of the regional differences in the prevalence of diabetes mellitus, hypertension and obesity can be explained through differences in age, diet, physical exercise, psycho-social discomfort, lack of social support, educational status, and migration background, a large proportion of this variance remains yet unexplained. It would be of great importance to public health and preventive measures to take a closer look at spatial differences in CVD morbidity and mortality to better tailor programmes to the regional environments and settings.

## Competing interests

The authors declare that they have no competing interests.

## Authors' contributions

The work presented here was carried out in collaboration between all authors. TED developed the idea for the article and carried out statistical analysis. KVS, AR and TED co-designed the theoretical background and interpreted the results. KVS researched and discussed the literature. KVS and TED co-designed and wrote the paper. All authors have contributed to, seen and approved the manuscript.
